# Association of Prenatal Alcohol Exposure and Prenatal Maternal Depression with Offspring Low-Grade Inflammation in Early Adolescence

**DOI:** 10.3390/ijerph18157920

**Published:** 2021-07-27

**Authors:** Janina Maschke, Jakob Roetner, Sophia Bösl, Anne-Christine Plank, Nicolas Rohleder, Tamme W. Goecke, Peter A. Fasching, Matthias W. Beckmann, Oliver Kratz, Gunther H. Moll, Bernd Lenz, Johannes Kornhuber, Anna Eichler, IMAC-Mind-Consortium

**Affiliations:** 1 Department of Child and Adolescent Mental Health, University Hospital Erlangen, Friedrich-Alexander University Erlangen-Nürnberg, 91054 Erlangen, Germany; Jakob.Roetner@uk-erlangen.de (J.R.); Sophia.Boesl@web.de (S.B.); Anne-Christine.Plank@uk-erlangen.de (A.-C.P.); Oliver.Kratz@uk-erlangen.de (O.K.); Gunther.Moll@uk-erlangen.de (G.H.M.); Anna.Eichler@uk-erlangen.de (A.E.); 2 Department of Psychology, Friedrich-Alexander University Erlangen-Nürnberg, 91052 Erlangen, Germany; nicolas.rohleder@fau.de; 3 Department of Obstetrics and Gynecology, University Hospital Erlangen, Friedrich-Alexander University Erlangen-Nürnberg, 91054 Erlangen, Germany; tamme.goecke@ro-med.de (T.W.G.); Peter.Fasching@uk-erlangen.de (P.A.F.); Matthias.Beckmann@uk-erlangen.de (M.W.B.); 4 Department of Obstetrics and Gynecology, RoMed Klinikum Rosenheim, 83022 Rosenheim, Germany; 5 Department of Psychiatry and Psychotherapy, University Hospital Erlangen, Friedrich-Alexander University Erlangen-Nürnberg, 91054 Erlangen, Germany; bernd.lenz@zi-mannheim.de (B.L.); Johannes.Kornhuber@uk-erlangen.de (J.K.); 6 Department of Addictive Behavior and Addiction Medicine, Central Institute of Mental Health (CIMH), Medical Faculty Mannheim, Heidelberg University, 68159 Mannheim, Germany

**Keywords:** prenatal alcohol exposure (PAE), meconium ethyl glucuronide (EtG), maternal self-report, prenatal depression, pregnancy, low-grade systemic inflammation, high-sensitivity C-reactive protein (hsCRP), early adolescence

## Abstract

(1) This longitudinal study aimed to investigate the link between prenatal alcohol exposure and prenatal maternal depression with the offspring’s low-grade inflammatory status. (2) Prenatal alcohol exposure was determined via maternal self-report during the 3rd trimester of pregnancy (self-report+: *n* = 29) and the meconium alcohol metabolite Ethyl Glucuronide (EtG), collected at birth (≥30 ng/g: *n* = 23). The Edinburgh Postnatal Depression Scale (EPDS) was used to screen for prenatal maternal depressive symptoms during the 3rd trimester (≥10: *n* = 35). Fifteen years later, 122 adolescents (*M* = 13.32 years; 48.4% female) provided blood samples for the analysis of high sensitivity C-reactive protein (hsCRP; *M* = 0.91; *SD* = 1.28). (3) Higher hsCRP levels were found in EtG positive adolescents (*p* = 0.036, ηp^2^ = 0.04) and an inverse non-significant dose–response relation with hsCRP (*r* = −0.35, *p* = 0.113). For maternal self-reported prenatal alcohol consumption (*p* = 0.780, ηp^2^ = 0.00) and prenatal depressive symptoms (*p* = 0.360, ηp^2^ = 0.01) no differences for hsCRP levels between the affected and unaffected groups were found. (4) Adolescents with prenatal alcohol exposure are at risk for low-grade systemic inflammation. The EtG biomarker may be more accurate compared to self-reports. The findings suggest that prenatal maternal depression does not evoke low-grade systemic inflammation.

## 1. Introduction

During pregnancy, women experience changes in almost every aspect of their biological, mental, and social life [1,2]. Some maternal factors affect the developing child only for a short period, while other factors may lead to long-term consequences [3,4]. According to the Developmental Origins of Health and Disease (DOHaD) hypothesis, the embryonic or fetal organism anticipates the environment after birth based on signs from the mother and adapts its phenotypical development to this information [5].

The human immune system starts to develop during the early stages of pregnancy. It can be divided into the innate and the adaptive immune system. As the activation of the innate immune system is the first response to tissue damage or microbial invasion, its markers can be used to get an impression of the organism’s health. One such biomarker for inflammatory processes is C-reactive protein (CRP) [6]. CRP is responsible for triggering the activation of the complement system as well as for promoting phagocytosis and the production of cytokines via activation of neutrophils and monocytes [7,8]. Four to six hours after an infection or injury, the CRP level rises up to 1000 times higher than baseline concentration [9]. Depending on the infection or injury, the immune response usually ends after several days or weeks [8]. The former infection or injury stops triggering an immune response if it was successfully fought, while anti-inflammatory agents get sent out by the immune system to actively suppress the immune response [10]. Opposing to immune reactions to an acute stimulus, systemic low-grade inflammation is not triggered by injuries or infections, but rather caused by global and chronic physical or psychological stressors [11]. These low-grade inflammatory processes last longer than acute inflammatory responses and are not localized in specific regions of the organism. They may occur in seemingly healthy individuals who do not suffer from any acute infections or injuries [12].

Traditional CRP assays have a lower limit of detection, for this reason high-sensitivity CRP (hsCRP) assays have been established to combat this issue [13]. hsCRP can be detected in blood plasma, blood serum, as well as in small blood spots from capillary or venous blood on filter paper (dried blood spots method, DBS) [14]. However, plasma and serum samples have revealed higher hsCRP values compared to samples derived from DBS [15]. DBS is minimally invasive and only a small quantity of blood is required for analysis; this enables collection of hsCRP samples in children. HsCRP proves to be a valuable biomarker, that is linked to certain health conditions, for example cardiovascular diseases and obesity. In clinical practice, beyond others, hsCRP is used to identify at-risk patients for coronary heart diseases [16]. Several studies link systemic low-grade inflammation to a heightened risk for developing coronary heart diseases, dying from coronary heart disease, or having an ischaemic stroke, especially in middle-aged men [17,18]. Other studies have also found an association of elevated (hs)CRP levels with overweight and obesity in adults as well as in children [19,20,21,22,23]. Elevated (hs)CRP levels have also been associated with mental health issues, e.g., to increased risk for depression or psychological distress [24].

Alcohol consumption during pregnancy is also considered a risk factor for child development. Only a few animal studies have examined the possible association between prenatal alcohol exposure (PAE) and elevated maternal (hs)CRP levels. The global prevalence of consuming alcohol during pregnancy is assumed to be around 10% [25]. Alcohol is a teratogen which crosses the placenta and directly affects the embryo or fetus [26]. Due to the inability to process alcohol properly, the embryonic or fetal organism is exposed to the harmful effects of alcohol significantly longer than the mother [27]. It is known that many aspects of the innate immune response and the mechanisms terminating the innate immune response are influenced by alcohol [28,29]. The exact effect of alcohol on the immune response is dependent on the actual blood level of alcohol, previous patterns of alcohol consumption, and the current dose and timing of alcohol consumption [29]. As the innate immune system develops continuously during gestation [30], a direct impact of alcohol on the fetal immune system is assumed [31]. Most of the observed consequences of PAE on the inflammatory system are known from animal testing [32]. The effect of PAE on the human immune system has not been fully investigated yet. In a small sample study, Ahluwalia et al. reported an increased concentration of pro-inflammatory cytokines in both mother and child when the mother reported to have consumed more than 60 drinks per month during pregnancy, compared to mothers who drank less or were abstinent during pregnancy [33]. Bodnar et al. [34,35,36] found that certain clusters of cytokines are activated when alcohol is consumed prenatally, both in the mother and in the offspring. They also reported increased CRP concentrations in mothers with heavy or moderate alcohol consumption during pregnancy and low CRP levels in mothers with low or no consumption [35].

Mental health issues during pregnancy may also contribute to a change in the maternal immune response. Depression is one of the most prevalent mental illnesses, also during pregnancy with a prevalence between 6 to 38% [37,38], and is linked to increased inflammation (Raison and Miller, 2011). Prenatal depressive symptoms are a risk factor for child development and are associated with e.g., birth outcomes, poorer regulation skills and slower habituation, cognitive developmental delay, and mental health problems [39,40,41,42]. This relationship is evident both for childhood [43,44] and for adolescence [45,46]. Prenatal maternal depression and its impact on the immune response of the mother and offspring has been scarcely investigated. Research indicates a link between maternal depression and inflammation during pregnancy as described in the review by Christian [47]. Plant and colleagues associated depression during pregnancy with an over-activation of the maternal immune system and found an association of maternal prenatal depression with elevated offspring hsCRP at age 25 [48]. Until now, no study investigated the effect of prenatal maternal depressive symptoms on offspring’s inflammation activity in adolescents.

Until now, most studies reporting on altered CRP levels in the child after intrauterine alcohol exposure were based on animal models. In the present study, we are to our knowledge the first to examine the association of meconium EtG and self-reported alcohol intake with hsCRP levels in adolescents.

In summary, the present study investigates the association of prenatal intrauterine alcohol exposure and prenatal maternal depressive symptoms respectively with altered hsCRP levels in adolescence. The study aims to further investigate the relation between PAE/maternal prenatal depression and a systemic state of low-grade inflammation in the offspring during adolescence.

## 2. Materials and Methods

### 2.1. Study Design

**Figure 1:**
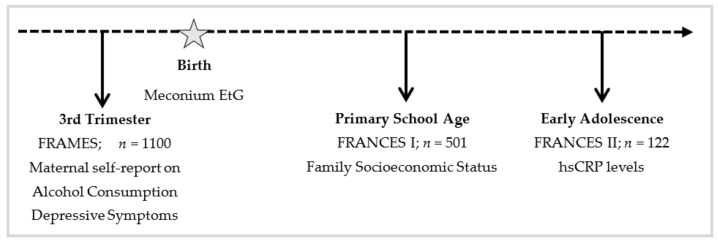
Timeline for Study Design.

The present study includes data of pregnant women from the FRAMES study (depicted in Figure 1 (Franconian Maternal Health Evaluation Studies; during pregnancy) [49,50], a collaboration of the Department of Obstetrics and Gynecology and the Department of Psychiatry and Psychotherapy at the Friedrich-Alexander-Universität Erlangen-Nürnberg, Germany. Offspring data were derived from the first and second follow-up trials, FRANCES and FRANCES II (Franconian Cognition and Emotion Studies; during childhood and adolescence) [51], which were conducted by the Department of Child and Adolescent Mental Health at the Friedrich-Alexander Universität Erlangen-Nürnberg in Erlangen.

For FRAMES, a total of 1100 women were recruited (years 2005–2007). The women were outpatients at the Obstetrics and Gynecology Department of Friedrich-Alexander University Erlangen-Nürnberg. To participate in FRAMES, the women had to be of legal age (≥18) and needed to be past the 30th week of pregnancy. Data were collected pre-, peri-, and postnatally (during the 3rd trimester, in the delivery room within 24 h after birth and 6–8 months after birth). The women’s depressive symptoms and alcohol consumption during gestation were assessed via self-report in a structured face-to-face interview conducted by a trained interviewer during the 3rd trimester of pregnancy. Birth outcomes of the children were registered immediately after delivery. The child’s meconium was collected within the first 24 h after birth and frozen at −80 °C.

From 2012 to 2015, *n* = 501 randomly selected mothers who had participated in FRAMES were invited to be part of FRANCES [51]. At-risk participants (including prenatally depressed mothers or neonates with detectable EtG values in the meconium) were underrepresented in the initial sample. To adjust for this, a pre-planned oversampling of at-risk women (*n* = 117) was carried out. Of the resulting 618 mother–child dyads, 245 families (39.6%) participated in FRANCES. FRANCES aimed to investigate prenatal influences on the child’s emotional, cognitive, and social development at the age of 6 to 9 [51,52,53,54,55]. When comparing women who participated in FRANCES I to women who did not take part in the study, no differences in maternal education (χ^2^(1) = 0.08, *p* = 0.774), marital status (χ^2^(1) = 0.16, *p* = 0.690), or family income (χ^2^(2) = 0.97, *p* = 0.616) were observed. All participants of FRANCES I were asked to re-participate in FRANCES II. FRANCES II (2019–2021) is the second follow-up study of FRAMES to examine the prolonged impact of pre-, peri-and postnatal influences on the offspring’s emotional, cognitive, and social development at the age of 11 to 14. 186 (75.9%) of the 245 contacted families (*n* = 188 children, due to two pairs of twins) agreed to re-participate (child age: *M* = 13.32, *SD* = 0.34, range 12.79–14.38). 167 families (89.8%) participated in person and 21 families (10.2%) only filled out questionnaires by post. A total of 32 families (13.1%) did not want to continue the participation, another 27 families (11.0%) were not reachable. When comparing participating families with non-participating families, no differences in marital status (χ^2^(1) = 0.35, *p* = 0.552), family income (χ^2^(4) = 3.94, *p* = 0.414), or maternal total psychopathology (*t*(234) = −0.93, *p* = 0.353) at time of FRANCES I were found. However, higher educated mothers were more likely to re-participate (χ^2^(1) = 7.60, *p* = 0.006).

The study was approved by the Local Ethics Committee and was conducted in compliance with the Declaration of Helsinki. Parental informed consent as well as adolescent assent was obtained from all participants.

### 2.2. Sample Characteristics

Of the 186 families with 188 children that contributed data for FRANCES II, 147 (78.2%) adolescents provided blood specimens for hsCRP quantification. Data from adolescents whose hsCRP level indicated a current inflammatory process (≥10 mg/L; *n* = 4), who took anti-inflammatory drugs the day of data collection (*n* = 11) or who drank at least one unit of alcohol in the last seven days (*n* = 1) were excluded from the analysis. Twins were also excluded from the analysis (two pairs, *n* = 4 children) as their intrauterine environment is not comparable to singleton pregnancies. Data from *n* = 4 adolescents were excluded as their mothers smoked heavily (≥five cigarettes/day) during pregnancy. No adolescent smoked in the last seven days before the appointment. Finally, outliers with an hsCRP level that was four standard deviations above the mean were also eliminated (*n* = 2).

Therefore, data from *n* = 122 hsCRP specimens were used in the present study (range: 0.02 mg/L to 6.04 mg/L). Out of these 122 adolescents, meconium was collected and EtG was determined in *n* = 104 (range: 0 ng/g to 2400 ng/g) at the time of birth. Self-report on alcohol consumption (range: yes to no) and depressive symptoms during pregnancy (range: 0 to 22) was obtained from all participants.

EtG above the cut-off (≥30 ng/g, EtG_30_+) was found in 22.1% (*n* = 23) of the collected specimens (*M_EtG_* = 418.22 ng/g; *SD_EtG_* = 613.59 ng/g) (Table 1). 23.8% (*n* = 29) of the participating women confirmed consuming alcohol during pregnancy (via maternal self-report, self-report+), most of them (*n* = 28) stated that they drank rarely during pregnancy. 76.2% (*n* = 93) women reported no drinking while being pregnant (self-report−). 28.7% (*n* = 35) of the women showed symptoms of prenatal depression (EPDS ≥ 10), with an average EPDS score of 12.49 (*SD_EPDS_* = 2.95), *n* = 5 women experienced symptoms of prenatal depression and had an EtG above the cut-off (≥30 ng/g, EtG_30_+). 71.3% (*n* = 87) of the women did not meet the cut-off criteria (*M_EPDS_* = 3.97; *SD_EPDS_* = 2.51).

The participating adolescents were 12.79 to 14.38 years old (*M_age_* = 13.32 years, *SD_age_* = 0.33). Most of the adolescents were attending twelve years of schooling (69.7%; *n* = 85), 26.2% (*n* = 32) of the adolescents were attending schools with ten years of schooling, 4.1% (*n* = 5) of the adolescents were attending schools with eight years of schooling. Most of the adolescents (70.5%, *n* = 86) were in 7th grade at the time of data collection. *n* = 29 adolescents (23.8%) were in 8th grade and *n* = 7 adolescents (5.7%) were in 6th grade. Further sample characteristics are presented in Table 1.

### 2.3. Instruments and Measures

#### 2.3.1. Prenatal Alcohol Exposure

EtG was determined as part of FRAMES. Meconium can be used to detect prenatal alcohol exposure in the third trimester. The accumulation of meconium EtG starts at week 20 of gestation until birth. One gram of meconium was collected 2 to 24 h after birth and frozen at −80 °C for up to 30 months until analysis. The whole procedure for collecting, storing, and analysing the EtG samples is described in detail by Bakdash et al. [56]. The recognized limit of detection for EtG is 10 ng/g [56]. However, the value to classify PAE varies from study to study 10 ng/g [57], 30 ng/g [53,58], 120 ng/g [50], and 154 ng/g [55]. For this study, 30 ng/g was used, because the sample size allowed this cut-off for detection, while a higher detection limit would have resulted in a sample size that was too small.

#### 2.3.2. Maternal Self-Report on Prenatal Alcohol Consumption

Maternal prenatal alcohol consumption was assessed in FRAMES during the third trimester of pregnancy. Participants were asked about their drinking behaviour during the current pregnancy in a face-to-face interview (No, I do not drink in general; No, I did not drink during pregnancy; Yes, I rarely drank during pregnancy; Yes, I drank one glass/day during pregnancy; and Yes, I drank more than one glass/day during pregnancy) (the frequencies are reported in [51]). No questions were asked regarding the exact number of drinks the women had consumed, or if the drinking behaviour changed during pregnancy [51]. Most participants, who reported that they consumed alcohol during pregnancy, stated that they “rarely” consumed alcohol. Only one of the participants answered, “Yes, I drank more than one glass per day during pregnancy”. For data analysis two groups were designed: Drinking (“I rarely drank during pregnancy” + “I drank one glass/day during pregnancy”) vs. no drinking (“I don’t drink in general” + “I didn’t drink during pregnancy”).

#### 2.3.3. Prenatal Depressive Symptoms

Prenatal depressive symptoms were assessed in FRAMES during the 3rd trimester of pregnancy via the Edinburgh Postnatal Depression Scale [59,60]. The EPDS consists of ten self-report questions about the presence and intensity of the mother’s affection and cognition during the last seven days (e.g., “I felt anxious and was worried without reason”, “I was sad and felt miserable”) [59]. The EPDS is also validated for being used antepartum [61], with possible restrictions in the test’s sensitivity [62]. Each of the ten items is rated from 0 to 3, resulting in a possible maximum score of 30. In general, higher scores indicate more symptoms. The scores can also be categorized by cut-off values: women with scores from 10 to 12 are considered to show mild depressive symptoms while scores above 12 indicate moderate to grave depressive symptoms [49,59,61]. In this study, a score of 10 or higher was interpreted as having depressive symptoms.

#### 2.3.4. Assessment of High-Sensitivity CRP

The adolescent’s hsCRP levels were assessed in FRANCES II. The concentration of hsCRP was analyzed in at least one drop of capillary blood. A sterile Safety-Lancet Extra 18 G with a penetration depth of 1.8 mm (Sarsted Salivetten^®^, Sarstedt, Nümbrecht, Germany) was used for pricking the participant’s finger after cleansing it with 70% EtOH. The blood was applied to Protein Saver Snap Apart filter paper (Whatman™, Maidstone, UK). After at least eight hours of drying at room temperature, the specimens were packed into Multi Barrier Pouches, (Size 4.375” × 8” O.D., Whatman™, Maidstone, UK) together with a 0.5 g silica gel sachet (Celloexpress, Antrim, UK) and stored at −80 °C until analysis. A CRP high sensitive enzyme linked immunosorbent assay (hsCRP-ELISA; IBL International, Hamburg, Germany) was used for the quantitative high sensitive determination of CRP levels. First, a 3.5 mm core was punched out of the filter paper using a Biopunch^®^ hand punch (Plano, Wetzlar, Germany) and each sample was transferred into one well of a 96-well plate (Sarstedt, Nümbrecht, Germany). After an overnight elution at 4 °C in 250 μL phosphate buffered saline (Roth, Karlsruhe, Germany) containing a complete protease inhibitor cocktail (Roche, Basel, Switzerland) and 0.1% Tween 20 (Roth, Karlsruhe, Germany), the samples were incubated for one hour at room temperature on a microplate shaker (300 rpm).

hsCRP levels in 100 µL of eluate were measured following the manufacturer’s instructions. Briefly, 10 × prediluted standard sera with different hsCRP concentrations provided by the ELISA kit were diluted 1:100 with sample diluent. All standard and blood samples were assayed in duplicate; optical density was determined at 450 nm using a microplate reader (Benchmark Plus microplate reader, BioRad, Hercules, CA, USA). The samples’ hsCRP levels were quantified against a standard curve (generated via five-parameter logistic curve fit) and the average value of each duplicate measurement was used in subsequent analyses. The intra- and inter-assay coefficient of variation was 6.3% and 6.9%, respectively; analytical sensitivity was approximately 0.02 mg/L.

### 2.4. Covariates

The socioeconomic status was calculated on the basis of parental education (4 = 12/13 years of schooling; 3 = 10 years of schooling; 2 = 9 years of schooling; 1 = <9 years of schooling) and family income (6-levels: <1000 Euro/month to >5000 Euro/month) (sum-index theoretical range: 3–14).

### 2.5. Confounders

When analysing inflammatory processes, the following variables that potentially interact with the inflammatory process were tested: Birth weight (gram), maternal age at birth, and the perinatal health of the neonate assessed via APGAR-scores, body-mass-index (BMI, adolescents were weight and measured in FRANCES II), the offspring’s sex (assigned at birth), child age (years), migration background, sleeping patterns, anti-inflammatory medication, alcohol, and tobacco use.

APGAR Score: Immediately after birth, three APGAR Scores were registered in the maternity room (infant’s heart rate, breathing efforts, reflexes, muscle tone, and colour). The scores were recorded 1, 5, and 10 min after birth [63]. A score of 10 represents the best possible condition. For this study, the three APGAR scores were averaged.

Migration Background: All children were born in Erlangen, Germany, only the parental birth countries were crucial for determining the offspring’s migration background. No distinction was made between adolescents with one migrated parent and adolescents with both parents born in another country.

Sleeping Pattern: Information regarding the adolescents sleeping pattern was collected using the Pittsburgh Sleep Quality Index [64]. No adolescent stated sleep disturbance in the last week prior to assessment. Therefore, no further participants were excluded [65].

Anti-Inflammatory Medicine: All mothers were asked about regular intake of child medication. The following medication was taken into account: hormonal contraception, systemic or respiratory steroids, anti-depressants or hormonal growth medication, as well as antihistamines, antibiotics, or medicine that eases an acute inflammation [48,66]. Additionally, medicine with a half-life shorter than one day was only considered if taken on the day of blood collection.

Alcohol and Tobacco: Adolescents were asked about their alcohol consumption (“Have you ever consumed any type of alcohol?”; “Have you consumed any type of alcohol in the last 12 months?”; “Have you consumed any type of alcohol in the last 30 days?”; “How old were you when you consumed alcohol for the first time?”; “How old were you when you were drunk for the first time?”). If the participants stated yes, further follow back was done: They were asked when and how many units of alcohol they consumed in the last 30 days. Adolescents who drank at least one unit of alcohol in the last seven days before the appointment were excluded from the analysis (*n* = 1). Statements about having one sip of alcohol were considered as no drink. Similar to alcohol consumption, the adolescents were asked about smoking cigarettes, hookah, and marijuana (“Have you ever smoked cigarettes/hookah/marijuana?”; “Have you smoked cigarettes/hookah/marijuana in the last 12 months?”; “Have you smoked cigarettes/hookah/marijuana in the last 30 days?”; “How old were you when you smoked cigarettes/hookah/marijuana for the first time?”). Adolescents who smoked at least in the last seven days before the appointment were excluded from the analysis. No adolescent stated to have smoked in the last week prior to assessment. No further questions were asked considering the amount of smoking.

### 2.6. Statistical Analysis

The analyses were carried out using IBM SPSS Statistics Version 21.0 (IBM Corp, Armonk, NY, USA). In order to form dichotomous variables and run group comparisons, EtG levels were classified in EtG_30_+ for EtG levels ≥30 ng/g and EtG_30_− for EtG levels <30 ng/g. Maternal self-report was grouped in self-report+ (confirmed drinking during pregnancy) and self-report− (denied drinking during pregnancy). EPDS scores were grouped in scores equal to or greater than 10 (EPDS ≥ 10) and less than 10 (EPDS < 10). Means (*M*), standard deviations (*SD*), and frequencies (*n*) were reported for hsCRP values and sample characteristics. Due to missing data regarding some sample characteristics (due to incomplete information from either mother or offspring), the sizes of the analyzed samples vary. Different sample sizes are indicated in the notes to the respective tables.

For all analyses, the probability of error was α = 5% as the level of significance was defined as *p* < 0.05 (two-tailed). *p* < 0.01 was interpreted as highly significant and *p* < 0.10 was interpreted as a trend. To test for the assumption of homogeneity of variance, Levene’s test was used and if the assumption was not met, degrees of freedom (*df*) were adjusted. For each variable, first, uncontrolled differences between the groups regarding hsCRP values and sample characteristics were analyzed using *t*-tests and χ^2^ tests. *p*-values were reported as well as Cohen’s d or phi coefficient (φ) in absolute numbers for effect size estimations. Cohen’s d ≥ 0.20 was interpreted as a small effect, ≥0.50 as an intermediate effect and ≥0.80 as a large effect [67]. For the phi coefficient, |r| ≥ 0.10 was interpreted as weakly, |r| ≥ 0.30 was interpreted as mildly and |r| ≥ 0.50 was interpreted as strongly correlated. As hsCRP levels as well as EtG values were not normally distributed (hsCRP: Shapiro Wilk W(104) = 0.67, *p* < 0.001, EtG: Shapiro Wilk W(122) = 0.45, *p* < 0.001).), log-transformed (log10) data was used for further analysis.

Associations between hsCRP values and possible covariates were examined using Pearson’s correlations (r) and *t*-tests. Outcomes with |r| ≥ 0.10 were interpreted as weak, |r| ≥ 0.30 were interpreted as mildly associated, and |r| ≥ 0.50 were interpreted as strongly correlated [67]: If a sample characteristic was significantly associated with log- transformed hsCRP values (*p* < 0.05) and there were no treatment group differences in this variable [68].

Three analyses of covariance (ANCOVA) were conducted using the independent variables EtG_30_+ vs. EtG_30_−, self-report+ vs. self-report− and EDPS < 10 vs. EPDS ≥ 10 to identify significant differences in hsCRP levels between each group (in three separate analyses). Homogeneity of variance was asserted for each ANCOVA using Levene’s tests. Relevant confounders were controlled as covariates. F-values and *p*-values were reported. For effect size estimations, partial eta squared (η^2^) was also reported. η^2^ ≥ 0.01 can be interpreted as small effect, η^2^ ≥ 0.06 represents a medium effect size and η^2^ ≥ 0.14 is interpreted as a great effect [67]. A post-hoc power analysis to check for achieved main-effect (1-β) was performed by the statistic software G*Power [69].

In addition, dose–response effects were tested within the EtG_30_+ group and the EPDS ≥10 group by partial-correlations (r_p_) with controlled confounders.

## 3. Results

### 3.1. Descriptive Data

Means and standard deviations of the sample’s characteristics—both for the total sample and for each factor group—are presented in Table 1.

In order to identify potential confounding variables, hsCRP levels, and several possible interfering factors were tested for associations. The analyses are presented in Table 2. Only the offspring’s current BMI, presented in percentiles, was significantly associated with the offspring’s hsCRP level (*r* = 0.39, *p* < 0.001) and was therefore used as a covariate in the following analysis. There were no factor group differences for BMI (EtG+/−: t(101) = −1.29, *p* = 0.201; self-report: t(119) = 0.29, *p* = 0.774; EPDS: t(119) = 0.08, *p* = 0.937). Therefore, child BMI was used as a covariate in the following analyses. The categorical associations between the prenatal risk factors were non-significant (EPDS+/− and EtG+/−: χ^2^(1) = 0.41, *p* = 0.524, Φ = 0.06; EPDS+/− and self-report+/−: χ^2^(1) = 0.39, *p* = 0.535, Φ = −0.06; EtG+/− and self-report+/−: χ^2^(1) = 2.28, *p* = 0.131, Φ = 0.15—analyses are presented in Table 3.

### 3.2. Prenatal Alcohol Exposure and the Offspring’s hsCRP Level

**Figure 2:**
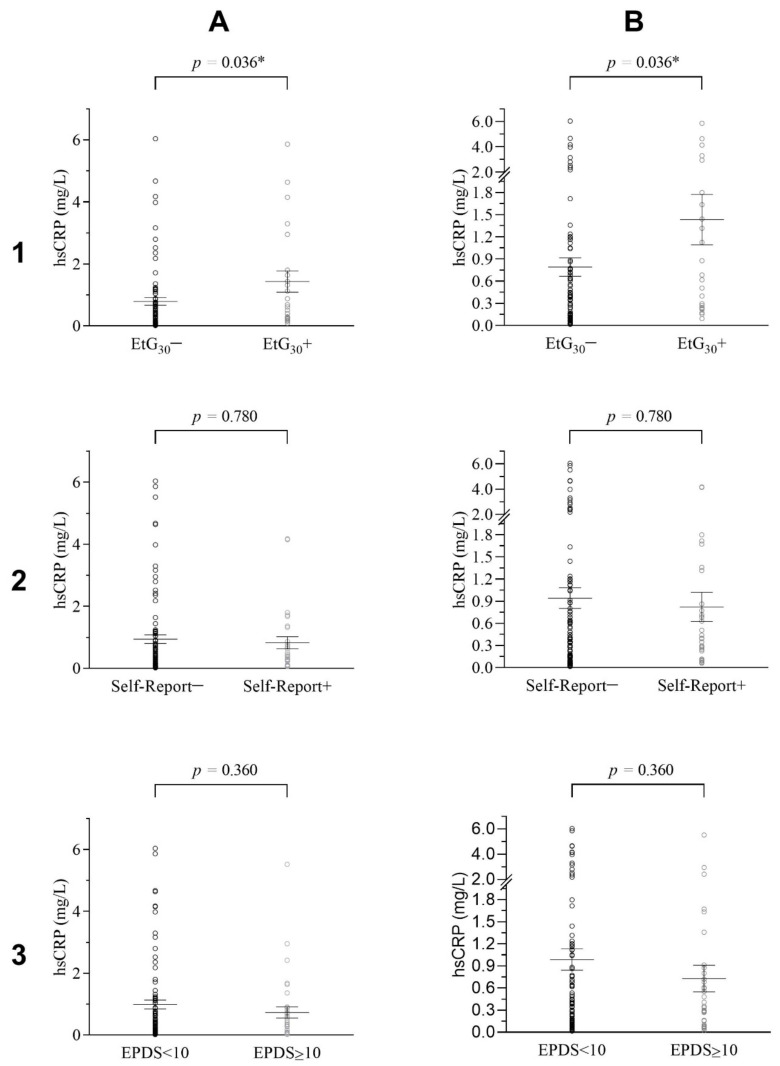
Distribution of Adolescent’s hsCRP Levels for EtG, Self-Report and EPDS Groups. (**1**), (**A**) Adolescent’s hsCRP levels grouped for EtG30+/EtG30− as an overview and (**B**) with focus on lower levels of hsCRP (*n* = 104). (**2**), (**A**) Adolescents hsCRP levels grouped for maternal self-report−/self-report+ on prenatal alcohol consumption and (**B**) with focus on lower levels of hsCRP (*n* = 122). (**3**), (**A**) Adolescents hsCRP levels grouped for EPDS < 10 / EPDS > 10 and (**B**) with focus on lower levels of hsCRP (*n* = 122). Error bars show standard errors; ANCOVA *p*-Levels assigned. * *p* < 0.05.

ANCOVA results for EtG_30_ indicated a significant difference in hsCRP values between the EtG_30_+ and the EtG_30_− group when controlling for the offspring’s BMI. Adolescents within the EtG_30_+ category (*M_hsCRP_* = 1.43, *SD_hsCRP_* = 1.63) showed higher hsCRP values than adolescents in the EtG_30_− category (*M_hsCRP_* = 0.79, *SD_hsCRP_* = 1.12), although the found effect size was small (*F*(1/100) = 4.52, *p* = 0.036, η_p_^2^ = 0.04), as depicted in Figure 2 and Table 4. For the test-power assuming a medium effect of η^2^ = 0.06, there was a test power of 1-β = 0.72 (EtG)/ 0.79 (for self-report), meaning there was a 72/79% probability to detect significant medium effects in the present sample. Assuming a small effect of η^2^ = 0.04, there was a test-power of 1-β = 0.42 (EtG)/0.48 (for self-report), meaning there was a 42/48% probability to detect significant small effects in the present sample.

**Figure 3:**
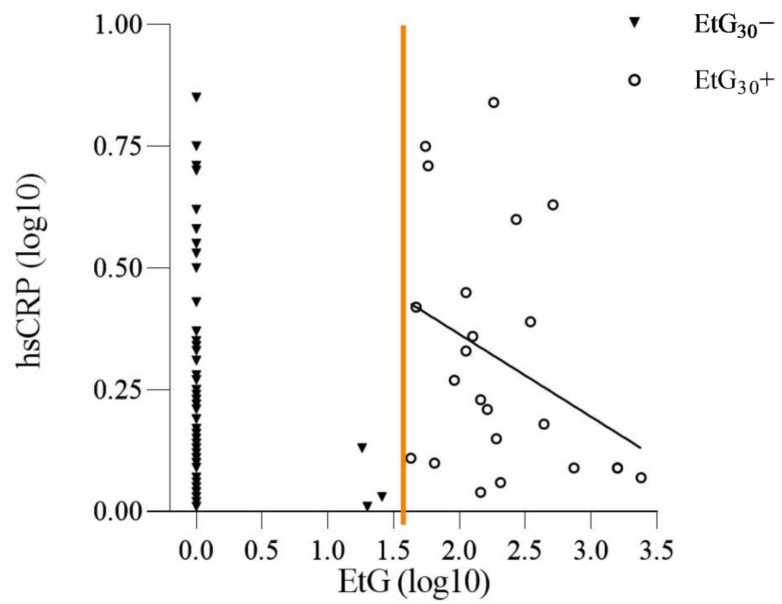
Dose–Response Relation of EtG Values and hsCRP Levels.

For EtG, the hypothesis of a dose–response effect of PAE on the adolescents hsCRP levels was tested in BMI-controlled partial-correlations. No significant dose–response association between EtG value and hsCRP level was found (*r* = −0.35, *p* = 0.113), as seen in Figure 3.

Distribution of the adolescents’ log-transformed (log10) high-sensitivity CRP (hsCRP) and their log transformed (log10) meconium ethyl glucuronide (EtG); EtG_30_+/EtG_30_−value at birth. Partialcorrelation of hsCRP levels and EtG values within the EtG_30_+ group (*n* = 23; *r* = −0.35, *p* = 0.113). Regression line depicts inverse dose–response relation of hsCRP levels and EtG values. Orange line indicates split between EtG_30_− and EtG_30_+.

For the second analysis, PAE was operationalized by the maternal self-report on prenatal alcohol consumption. The presumed difference in the adolescent’s hsCRP levels between the positive and negative maternal self-report group was tested via ANCOVA. As seen in Table 4 and Figure 2, no significant differences in the adolescents hsCRP levels were found between the self-report+ (MhsCRP = 0.82, SDhsCRP = 1.06) and the self-report− (MhsCRP = 0.94, SDhsCRP = 1.34) group, when controlling for the offspring’s current BMI (*n* = 121, F(1/118) = 0.08, *p* = 0.780, ηp^2^ = 0.00).

Overall, PAE was significantly associated with the offspring’s hsCRP level when using meconium EtG as in indicator for PAE. Maternal self-report on prenatal alcohol consumption was not associated with difference in hsCRP levels in the offspring.

### 3.3. Prenatal Maternal Depression and the Offspring’s hsCRP Level

Finally, an ANCOVA investigating a potential association between prenatal maternal depression and the child’s hsCRP level was conducted. Results indicated no significant difference in hsCRP values between EPDS < 10 (MhsCRP = 0.99, SDhsCRP = 1.35) and EPDS ≥ 10 (MhsCRP = 0.73, SDhsCRP = 1.07) groups when controlling for the offspring’s BMI (F(1/118) = 0.84, *p* = 0.360, ηp^2^ = 0.01), as seen in Table 4 and Figure 2. The EPDS test-power assuming a η^2^ = 0.06 medium effect was calculated; 1-β = 0.79, meaning there was a 79% probability to detect significant effects in the present sample. For assuming a small test-power of η^2^ = 0.04, there was a test power of 1-β = 0.48, meaning there was a 48% probability to detect significant small effects in the present sample.

A potential dose–response effect of prenatal maternal depression on the adolescents’ hsCRP levels was investigated using only data from participants within the EPDS ≥ 10 category (*n* = 35) for the analysis. No significant correlation between the adolescents hsCRP levels and maternal EPDS scores was revealed (*r* = −0.05, *p* = 0.792).

In summary, the analyses revealed neither a significant association nor a dose–response relation between prenatal maternal depression and the offspring’s hsCRP level in early adolescence.

## 4. Discussion

This study aimed to investigate the potential, long-lasting link of PAE as well as prenatal maternal depression on the offspring’s low-grade inflammatory processes. PAE was operationalized by applying maternal self-reports during the third trimester of pregnancy and by determining meconium EtG as a biomarker. The results revealed significantly increased hsCRP values in adolescents with meconium EtG (≥EtG_30_+) than in those below the threshold. Likewise, the dose–response relation between PAE and the offspring’s low-grade inflammation in early adolescence was examined. Higher EtG values above the cut-off were moderately but non-significantly correlated with lower hsCRP levels. The post-hoc power analyses presented a 72% probability for EtG and a 79% probability for maternal self-report to detect significant medium effects, close to the standard threshold of 80%. For assuming a small test-power the post-hoc power analyses presented a 42% (EtG)/ 48% (self-report) probability.

Firstly, the results indicate that intrauterine alcohol exposure predicts low-grade inflammation in early adolescence. These results support the hypothesis that alcohol causes changes in the maternal and embryonic immune response [28,29]. It can be assumed that PAE affects the embryo or fetus directly as a teratogen and indirectly via the maternal reaction [70,71]. Earlier research exhibits that adverse environmental factors during gestation may cause long-lasting changes to the immune system [36,72,73], which could be supported by the present study. In animal models, altered immune system development has already been observed due to PAE, causing an increase in CRP levels [36] and linking PAE directly to increased cytokine levels. Bodnar et al. (2018) exhibited disturbed cytokine levels in the PAE offspring directly after birth [35]. Our findings add to the knowledge that this association remains relevant into adolescence.

Nonetheless, it needs to be considered that a multitude of other factors—potentially related to PAE—may influence hsCRP levels in these adolescences, such as exposure to family violence, unhealthy diet, or socioeconomic status aspects. This is why we determined whether the EtG positive versus EtG negative groups differ in further characteristics, i.e., socioeconomic status, medication, alcohol, tabaco, migration background, sleep patterns, and BMI. Other factors besides intrauterine alcohol exposure may also play a role in positive EtG outcomes, which may be responsible for increased hsCRP values. However, not all associated explanations could be tested and should be focused on in future studies [34]. With reducing these confounders our results still present elevated CRP levels in the EtG positive group. Therefore, it can be assumed that long lasting negative health outcomes related to altered immune functions are specifically caused by PAE.

Secondly, additional focus was placed on the dose–response relation within the EtG positive group and hsCRP values. We were able to observe an inverse dose–response relation in the EtG+ group; however, these findings were not statically significant. There are several possible explanations for such a phenomenon. According to the DOHaD hypothesis changes in the maternal organism trigger adaptive processes in the embryonic and fetal development, for example, it has been observed that stress during pregnancy may be linked to an altered maternal immune system with consequences for the developing fetal immune system [74,75,76]. Interestingly, Antonson et al. observed decreased circulating maternal cytokine levels during psychological stress in pregnancy [77], which parallels our findings in PAE-offspring, where we found decreasing hsCRP levels with increasing EtG levels. Multiple researches indicate that prolonged exposure to stress can lead to hypocortisolism [78,79]. It may be speculated that a similar mechanism occurs with prolonged alcohol exposure and its immune stimulation. This would explain our dose–response findings. In addition to this, it is important to note that the dose–response relation in the EtG+ group could be attributed to the fact that only a small number of adolescents had respectively high EtG values, therefore a false positive test result needs to be considered too. If we would eliminate the three extremely high EtG+ values (EtG > 500 ng/g) of the three adolescents, the dose–response relation would not be present anymore (*r* = −0.11, *p* = 0.667).

In general, it was not possible for us to exactly determine the amount of alcohol consumed and its temporal extent regarding the whole course of pregnancy. Until now, few studies have attempted to link meconium EtG levels with concrete prenatal drinking amounts. In contrast, there are results for other meconium ethanol metabolites: fatty acid ethyl esters (FAEE) EtG and FAEE are highly correlated with each other [80]. Mothers of FAEE-positive children drank an average of 10 drinks/week and no fewer than four drinks/week [81]. One or two drinks per day were not enough to find positive FAEE meconium [82]. The EtG metabolite only illustrates the quantity of ethanol metabolite in meconium. We cannot precisely reflect how much of the ethanol metabolite came from alcohol or if it originated through other factors, e.g., specific food sources (e.g., fresh fruit juice) [80,83]. At last, it is also difficult to answer if zero alcohol was consumed in the EtG negative group, or if some amount of alcohol was consumed early during pregnancy, as EtG meconium only depicts PAE during the second and mostly third trimester of pregnancy, while the effects of consumption during early pregnancy remains unclear [56]. Consequently, we can only speculate how much alcohol was consumed during the early stages of pregnancy. The immune system already starts developing at the beginning of embryogenesis, so the harmful impact of early pregnancy exposure to alcohol on inflammatory processes needs to be assumed [84]. Still, research shows that alcohol consumption usually decreases during the course of pregnancy [85]. Therefore, we can assume that mothers of the positive EtG group also consumed alcohol in the beginning of pregnancy and that the statistically significant increased hsCRP values found in adolescents with meconium EtG (≥EtG_30_+) reflect continuous PAE during pregnancy. Until now, the relationship between drinking volume, drinking timing, and EtG levels in meconium has not been clarified. There is no safe-time-point or safe-amount for prenatal alcohol consumption.

The second operationalization of PAE was established via maternal self-report on prenatal alcohol consumption. In contrast to our findings on EtG and hsCRP levels, maternal self-reports did not reveal any significant association of PAE and the offspring’s hsCRP status. However, maternal self-reports on prenatal alcohol consumption are known to be inaccurate. Earlier work examined limited overlap between biomarker results of prenatal alcohol exposure and maternal self-reports [86], in a randomised sample, 15% of the EtG samples were above the threshold, however no mother reported heavy alcohol consumption during pregnancy [80]. Lange et al. also found the prevalence of EtG levels to be 4.26 times higher, compared to the maternal self-reports [87]. The reason for this inaccuracy may stem from a couple of reasons; women tend to underreport consumption due to social and memory biases, as already discussed in previous papers [51,88]. Additionally, it needs to be considered that the maternal self-report was collected retrospectively in the third trimester, therefore it only accounts for alcohol consumption for the first and second trimester, whereas EtG meconium is a biomarker only for the third trimester [56]. Moreover, studies have also displayed that multiple factors may influence the accuracy of the self-report i.e., social status or mothers’ age at pregnancy [89,90]. It was also exhibited that the wording of questioning, recall period, as well as displaying a list of drinks can influence the outcome of the maternal self-report [90]. In concordance with other researchers [86] we already displayed that the EtG biomarker was the more effective measure for predicting child development [55,57]. It is important to note that the EtG biomarker indicates intrauterine ethanol exposure, which is not linearly related to maternal alcohol consumption due to the individual metabolism of mother and foetus.

In summary, meconium EtG concentrations above the cut-off (EtG_30_+) were found to be associated with increased hsCRP levels. In addition, our results support earlier research that EtG as a biomarker of intrauterine alcohol exposure can be a useful predictor of child development. However, there is still abundant space for further research, since the mechanistic link between prenatal alcohol consumption and elevated hsCRP levels in the adolescent offspring is not clear. Future studies should explore the underlying pathways further and use a larger sample size, the larger sample size would also increase the testing power.

The other investigated factor with possible impact on the offspring’s low-grade inflammatory process was prenatal maternal depression. Contrary to expectations, this study located no statistical difference in hsCRP levels between adolescents of mothers who did suffer from symptoms of prenatal depression and those who did not. Likewise, no dose–response relation between maternal EPDS scores and the offspring’s hsCRP level in early adolescence was observed. The post-hoc power analyses presented a 79% probability to detect significant medium effects, and 48% to detect significant small effects.

In contrast to studies reporting on an association of depression and CRP levels [91], several studies also found that hsCRP was not elevated during depressive episodes in general or during episodes of prenatal maternal depression [92,93,94]. This suggests that no systemic low-grade inflammatory processes in the mother would be caused and no adoptive response in the embryonic or fetal organism would be triggered. Consequently, the fetus or embryo would not be affected by the maternal depressive episode. The present data cannot answer this question because there were no maternal hsCRP data during pregnancy available. Future longitudinal studies should continuously measure low-grade inflammation markers levels from the beginning of pregnancy until adolescence.

Another possible explanation for this might be that prenatal depression affects the maternal immune system including transmission processes to the fetal immune system, but only in a small subgroup of prenatally depressed women. Multiple studies reported signs of low-grade inflammatory processes in approximately one third of depressed individuals depending on the type of depression [95,96]. It can be argued that even if the adolescents have been impacted by the changed immune status of their mothers, the effect might not be detected due to the small number of adolescents concerned. Furthermore, due to the small sample size the cut-off for EPDS was set at 10, including both women with mild and severe symptoms, making a distinction between severe and mild symptoms impossible. The depressive symptoms were only collected once, however research indicates the EPDS score changes over time depending on the current mood of the participants [97]. Thus, false positive test results need to be considered also [62]. In summary, due to the way the EPDS was assessed in this study (cut-off at 10, single time assessment), no statement can be made concerning the severity of the prenatal depressive symptoms and its course. Other studies that used a clinical interview to diagnose a depressive episode during pregnancy found elevated hsCRP in the mother [98] or in the offspring years later [48]. Hence, it could be hypothesized that a variability of maternal depressive symptoms and more severe and extended depressive episodes during pregnancy have a greater impact on the maternal and fetal immune system, whereas milder symptoms might not be as influential. Therefore, future studies should investigate prenatal maternal depression and its relation to the immune system with a larger sample size, which would also increase the testing power, additionally the EPDS should be longitudinally assessed at all stages of pregnancy.

## 5. Conclusions

The present study is the first to investigate the association of PAE, using the biomarker meconium EtG, and prenatal maternal depression with hsCRP levels in adolescents. Our results show that prenatal alcohol exposure is related to a low-grade inflammatory process in adolescents. However, this can only be observed when prenatal alcohol exposure is operationalized via meconium EtG and not via maternal self-report. The study hereby further supports the notion that (1) even low consumption of alcohol during pregnancy is likely to exert adverse effects on the offspring and (2) self-reporting data needs to be carefully interpreted when it is used as the only source for depicting prenatal alcohol exposure and predicting child developmental outcomes. No effect of prenatal maternal depression on the offspring’s hsCRP concentration was found, which emphasizes the need to further research the overall link between depression and inflammation and to focus on severe courses of depression. We conclude: the awareness of the harmful effect of prenatal alcohol consumption for the developing child and the treatment of depressive symptoms during pregnancy is vitally important for maternal well-being, as well as for a favourable environment for the child.

## Figures and Tables

**Table 1:** Sample Characteristics and Mean/Frequency Comparison.

Parameters	Total *n* = 122	EtG *n* = 104					Self-Report *n* = 122					EPDS *n* = 122				
		EtG_30_− (*n* = 81)	EtG_30_+ (*n* = 23)				Self-Report− (*n* = 93)	Self-Report+ (*n* = 29)				EPDS < 10 (*n* = 87)	EPDS ≥ 10 (*n* = 35)			
	*M (SD)*	*M (SD)*	*M (SD)*	*t(df)*	*p*	*d*	*M (SD)*	*M (SD)*	*t(df)*	*p*	*d*	*M (SD)*	*M (SD)*	*t(df)*	*p*	*d*
EtG level	93.08 (332.77)	0.75 (3.92)	418.22 (613.59)	^c^ −29.71 (102)	0.000 **	1.47	106.43 (375.13)	48.58 (99.37)	^c^ −0.76 (102)	0.450	0.17	104.52 (363.62)	38.39 (77.46)	^c^ −0.42 (102)	0.679	0.21
EPDS score	6.41 (4.68)	5.44 (4.72)	6.26 (3.76)	−0.76 (102)	0.447	0.18	6.51 (4.60)	6.10 (5.02)	0.40 (120)	0.688	0.08	3.97 (2.51)	12.49 (2.95)	−16.09 (120)	0.000 **	3.23
hsCRP (mg/L) ^b^	0.91 (1.28)	0.79 (1.12)	1.43 (1.63)	^a^ 1.77 (102)	0.088 ^+^	0.52	0.94 (1.34)	0.82 (1.06)	−0.43 (120)	0.665	0.09	0.99 (1.35)	0.73 (1.07)	−1.00 (120)	0.317 *	0.20
Birth Weight	3446.23 (516.87)	3404.26 (443.24)	3681.74 (508.44)	−2.56 (102)	0.012 *	0.61	3405.64 (535.37)	3576.38 (435.58)	−1.56 (120)	0.121	0.33	3419.83 (456.88)	3511.86 (645.42)	^a^ 0.77 (120)	0.445 *	0.18
APGAR	9.36 (0.74)	9.40 (0.68)	9.38 (0.64)	−0.13 (100)	0.896 *	0.03	9.37 (0.74)	9.29 (0.76)	−0.47 (118)	0.639	0.11	09.44 (0.67)	9.14 (0.87)	^a^ −1.83 (118)	0.073 ^+^*	0.41
Age (Mother at Delivery)	32.52 (4.61)	32.73 (4.89)	32.26 (4.47)	−0.41 (102)	0.681 *	0.10	31.94 (4.69)	34.38 (3.89)	−2.54 (120)	0.012 *	0.61	33.05 (4.38)	31.20 (4.98)	−2.02 (120)	0.061 ^+^	0.41
Age (Offspring)	13.32 (0.33)	13.27 (0.29)	13.43 (0.39)	^a^ 1.89 (102)	0.068^+^	0.51	13.31 (0.32)	13.36 (0.36)	−0.67 (120)	0.506	0.15	13.30 (0.32)	13.37 (0.34)	−1.11 (120)	0.269 *	0.22
BMI percentile	51.21 (30.01)	51.46 (28.59)	57.26 (31.48)	−0.84 (101)	0.404 *	0.20	50.92 (29.12)	52.10 (33.24)	−0.18 (119)	0.855	0.04	51.79 (29.51)	49.77 (31.61)	−0.33 (119)	0.739 *	0.07
SES index	12.02 (1.86)	12.09 (1.86)	11.91 (1.83)	–0.39 (102)	0.693 *	0.10	11.86 (1.93)	12.52 (1.53)	^a^ 1.89 (120)	0.063 ^+^	0.38	12.16 (1.84)	11.66 (1.90)	−1.36 (120)	0.177 *	0.27
	**Total** ***n* = 122**	**EtG_30_** ***n* = 104**					**Self-Report** ***n* = 122**					**EPDS** ***n* = 122**				
		EtG_30_− (*n* = 81)	EtG_30_+ (*n* = 23)				Self-Report− (*n* = 93)	Self-Report+ (*n* = 29)				EPDS < 10 (*n* = 87)	EPDS ≥ 10 (*n* = 35)			
	*n*	*n*	*n*	*Χ* ^2^ *(df)*	*p*	Φ ^d^	*n*	*n*	*Χ* ^2^ *(df)*	*p*	Φ ^d^	*n*	*n*	*Χ* ^2^ *(df)*	*p*	Φ ^d^
Sex assigned at birth (female/male)	59/63	36/45	14/9	1.94 (1)	0.164	0.14	47/46	12/17	0.74 (1)	0.389	0.08	37/50	22/13	4.13 (1)	0.042 *	0.18
Prenatal Maternal Smoking (yes/no)	10/112	8/73	2/21	0.03 (1)	0.865	0.02	8/85	2/27	0.09 (1)	0.770	0.03	10/77	0/35	4.38 (1)	0.036 *	0.19
Anti-Inflammatory Medicine (yes/no)	12/110	5/76	2/21	0.18 (1)	0.670	0.04	10/83	2/27	0.37 (1)	0.543	0.06	7/80	5/30	1.09 (1)	0.295	0.09
Migration Background (yes/no)	13/109	9/72	2/21	0.11 (1)	0.740	0.03	9/84	4/25	0.39 (1)	0.531	0.06	10/77	3/32	0.22 (1)	0.636	0.04

Notes: EtG: Ethyl Glucuronide. hsCRP: high sensitive CRP. ^a^. t-score correction based on Levene for unequal variances. ^b^. hsCRP log10 transformed. ^c^. EtG log10 transformed. Birth weight in grams. ^d^. absolute values are displayed. APGAR score: average of three scores, best adaption = 10. Maternal age at delivery in years. Child age in years. BMI: child’s current body-mass-index in percentiles. SES: Socioeconomic status: combination of maternal/paternal education level (4-level: < 9, 9, 10 or 13 years) and net family income (6-level: < 1000 to > 5000) (sum-index, theoretical range: 3–14). Prenatal smoking: maternal self-report 3rd trimester. Anti-inflammatory medicine intake child: maternal self-report of regular medicine intake with anti-inflammatory effect over the last months. Migration background: at least one parent not born in Germany. Different sample size due to missing data: APGAR (*n* = 120); BMI percentile (*n* = 121). ^+^ *p* < 0.10, * *p* < 0.05, ** *p* < 0.01.

**Table 2:** Correlations and Mean Comparisons between the Offspring’s hsCRP Levels and Potential Confounders.

Potential Confounders	hsCRP (mg/L)	
	*r*	*p*
Birth Weight	0.07	0.467
APGAR	0.05	0.606
Age (Mother at Delivery)	0.08	0.390
Age (Offspring)	−0.12	0.176
BMI	0.39	<0.001 **
SES	0.02	0.858
	*t*	*p*
Sex	0.07	0.945
Prenatal Maternal Smoking	0.75	0.458
Anti-Inflammatory Medicine	0.02	0.987
Migration Background	−0.36	0.723

Notes: hsCRP log10 transformed. APGAR score: child adaption immediately after birth. BMI: child’s current body-mass-index. SES: Socioeconomic status: combination of maternal/paternal education level. Prenatal smoking: maternal self-report yes = 1/no = 0, 3rd trimester. Anti-inflammatory medicine intake child: mother-report of regular medicine intake with anti-inflammatory effect over the last 6 months yes= 1/no = 0. Migration background: at least one parent not born in Germany yes = 1/no= 0. ** *p* < 0.01.

**Table 3:** Association between Self-Report, EtG, and EPDS.

Risk Groups	*n*	Self+	Self−	χ^2^(1) *p* φ
EtG+	23	8 (34.8%)	15 (65.2%)	2.28
EtG−	81	16 (19.8%)	65 (80.2%)	0.131
*n*		24	80	0.15
		EPDS+	EPDS−	
EtG+	23	5 (21.7%)	18 (78.3%)	0.41
EtG−	81	13 (16.0%)	68 (84.0%)	0.524
*n*		18	86	0.06
		EPDS+	EPDS−	
EPDS+	35	7 (20.0%)	28 (80.0%)	0.39
EPDS−	87	22 (25.3%)	65 (74.7%)	0.535
*n*		29	93	−0.06

Notes: EtG: Ethyl Glucuronide. EPDS: The Edinburgh Postnatal Depression Scale. Self: maternal self-report. Rows in percentage, *n* = 104.

**Table 4:** Analyses of Covariance for adolescent’s high-sensitive CRP Levels in the EtG and EPDS groups.

ANCOVA Models	F(df/df)	*p*	η_p_^2^
EtG_30_			
Total Model	10.49 (2/100)	<0.001 **	0.17
EtG_30_	4.52 (1/100)	<0.036 *	0.04
Current BMI	14.95 (1/100)	<0.001 **	0.13
Self-Report			
Total Model	10.88 (2/118)	<0.001 **	0.16
Self-Report	0.08 (1/118)	0.780	0.00
Current BMI	21.72 (1/118)	<0.001 **	0.16
EPDS			
Total Model	11.33 (2/118)	<0.001 **	0.16
EPDS	0.84 (1/118)	<0.360	0.01
Current BMI	21.54 (1/118)	<0.001 **	0.15

Notes: EtG: ethyl glucuronide collected at birth, cut-off at 30 ng/g (*n* = 103; EtG_30_−: *n* = 80, EtG_30_+ = 23). self-report: maternal self-report on prenatal alcohol consumption, assessed during third trimester of pregnancy (*n* = 121; self-report−: *n* = 92, self-report+: *n* = 29). EPDS: Edinburgh Postnatal Depression Scale, completed during third trimester of pregnancy (*n* = 121, EPDS−: *n* = 86, EPDS+: *n* = 35) [59]. Covariate: Child’s current BMI (body-mass-index). * *p* < 0.05, ** *p* < 0.01.

## Data Availability

The data presented in this study are available on request from the corresponding author.
